# Severe rickets in a young girl caused by celiac disease: the tragedy of delayed diagnosis: a case report

**DOI:** 10.1186/1756-0500-7-701

**Published:** 2014-10-08

**Authors:** Butheinah A Al-Sharafi, Shafiq A Al-Imad, Amani M Shamshair, Derhim H Al-Faqeeh

**Affiliations:** Department of Medicine, Sana’a University Medical School, Sana’a, Yemen; Department of medicine, University of Science and Technology hospital, Sana’a, Yemen

**Keywords:** Celiac disease, Rickets, Failure to thrive, Anemia

## Abstract

**Background:**

Celiac disease is a systemic immune mediated disease which usually presents with gastrointestinal symptoms, but it may present with extra gastrointestinal manifestations such as metabolic bone disease and failure to thrive. This may lead to a delay in the diagnosis.

**Case presentation:**

We present a 13 year old female from the middle east with an 8 year history of severe rickets causing multiple bone deformities leaving the child crippled with bowing of both of her arms and legs. The patient was also found to have growth failure, anemia and on further workup she was found to have celiac disease.

**Conclusion:**

We are presenting this case because it shows a severe case of rickets after malabsorption for many years. Celiac disease should be kept in mind as a cause of rickets in patients not responding to usual forms of treatment or when associated with other manifestations of malabsorption.

**Electronic supplementary material:**

The online version of this article (doi:10.1186/1756-0500-7-701) contains supplementary material, which is available to authorized users.

## Background

Celiac disease (CD) is recognized as a systemic immune mediated disorder triggered by dietary gluten in genetically susceptible persons. It may affect persons of any age and many races and ethnic groups. Frequent symptoms and signs include chronic diarrhea, weight loss, and abdominal distention (in 40-50% of patients) [1]. Other manifestations include iron deficiency anemia, osteoporosis. Less common presentations include abdominal pain, constipation, weight loss, neurologic symptoms, dermatitis herpetiformis, hypoproteinemia, hypocalcemia and elevated liver enzymes [1]. Rare presentations such as fever of unknown origin [2] and psychosis have been reported [3]. Osteomalacia [4, 5] and rickets [4, 6–8] may also be the presenting feature of celiac disease. We present a case of rickets in a 13 year old girl from the middle east that came to us with severe deformities in the extremities causing severe disability. She had a long history of multiple non-traumatic fractures including fractures of the arms causing severe bowing of the arms which is rarely seen in rickets. This patient was found to have CD. Early diagnosis and treatment in this case could have prevented the patient from becoming crippled.

## Case presentation

**Figure 1:**
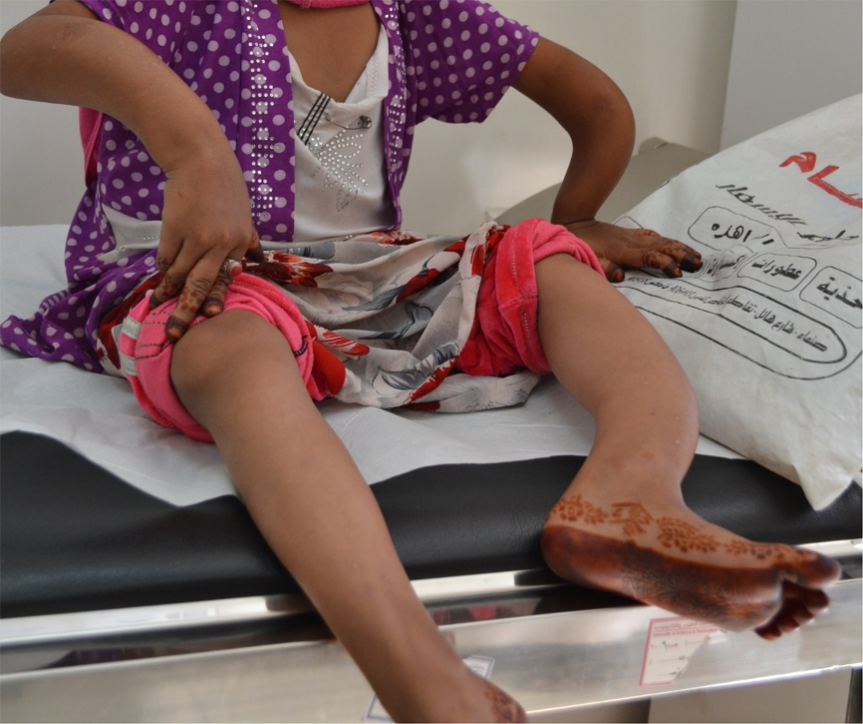
**Photo of the patient’s extremities showing severe growth failure and bowing of the arms and legs.**

A 13 year old female living in a remote rural area came to our clinic with an 8 year history of deformities in the extremities which had gradually became worse till she was unable to walk. The patient over the years had developed recurrent fractures in her legs and arms after minor falls. The family was poor and lived in a remote area far away from proper medical facilities. She was treated by local healers for her fractures which resulted in bowing of legs and arms. There were no gastrointestinal symptoms of abdominal pain or diarrhea. She was brought to the clinic carried by her father. The patient used her arms to drag herself around the house and developed fractures followed by severe bowing of her arms. She had been diagnosed with rickets and iron deficiency anemia by doctors in community hospitals and had received Vitamin D and iron supplements many times without improvement. She had never had a complete workup to find out the cause of her rickets. The patient also had failure to thrive. On examination the patient was pale, weight was 11 kg and height 97 cm (below the 3^rd^ percentile for her age) (Figure 1). She had severe bowing of her arms and legs.

Due to the presence of anemia, failure to thrive and rickets the initial impression was malabsorption probably due to celiac disease and the laboratory work up was done accordingly.

**Figure 2:**
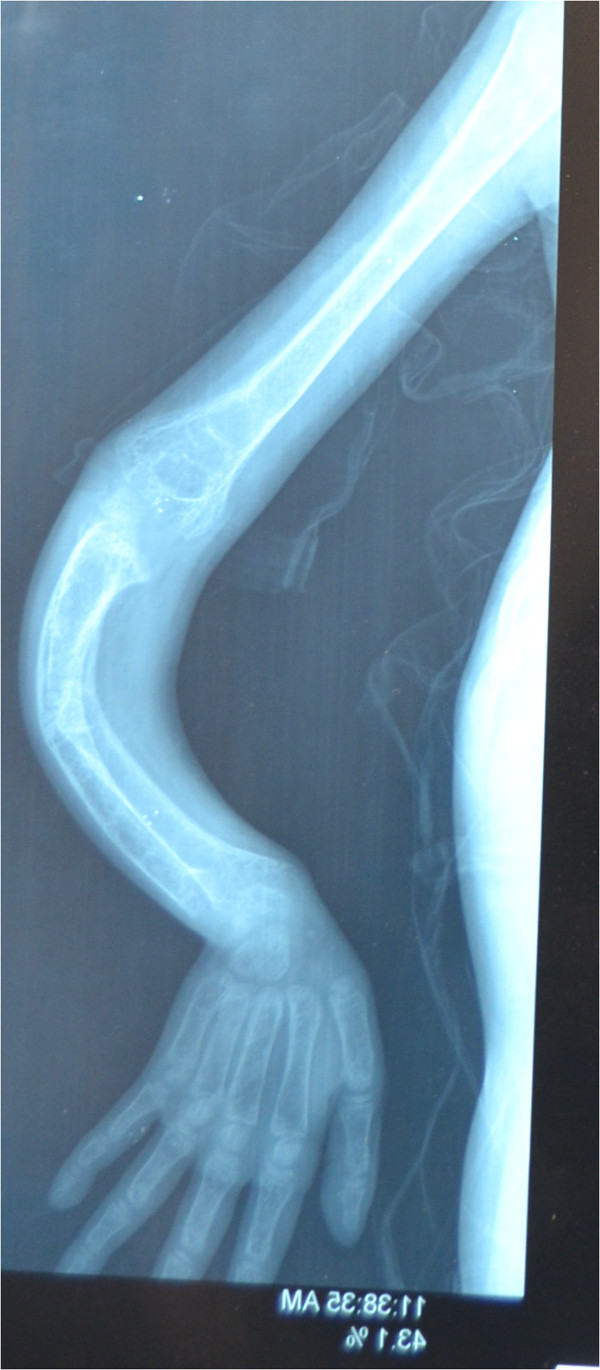
**X-ray of the patient’s upper limb showing diffuse osteopenia and bowing of forearm with blurring of the metaphyseal lines.** It also shows dense transverse lines in the ulna suggestive of looser’s zones indicative of severe rickets.

**Figure 3:**
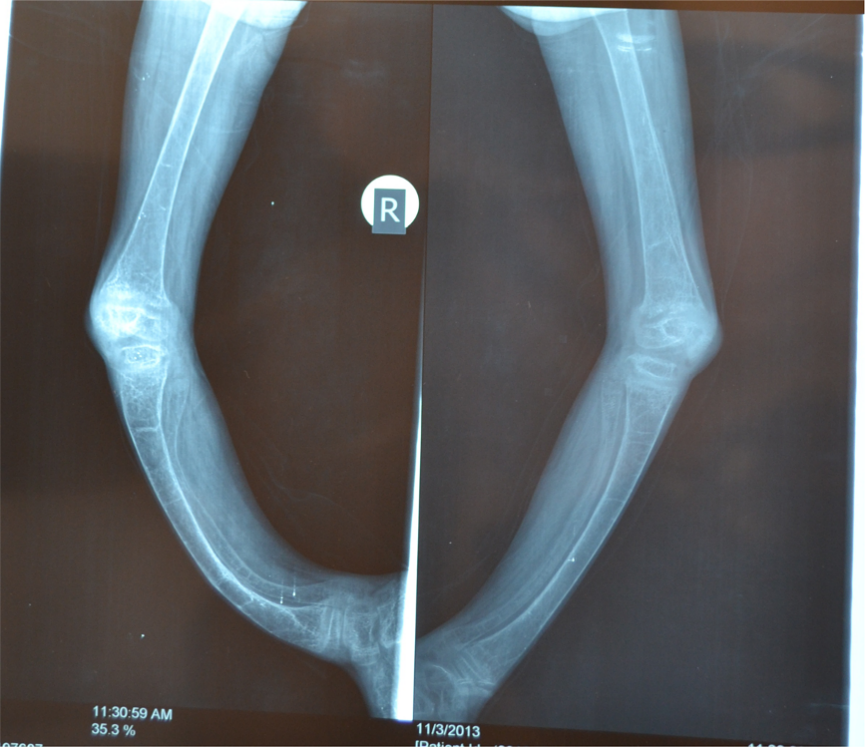
**X-rays of both lower limbs showing severe bowing of the legs and diffuse osteopenia.** It also shows dense transverse lines in the tibia suggestive of looser’s zones indicative of rickets.

**Figure 4:**
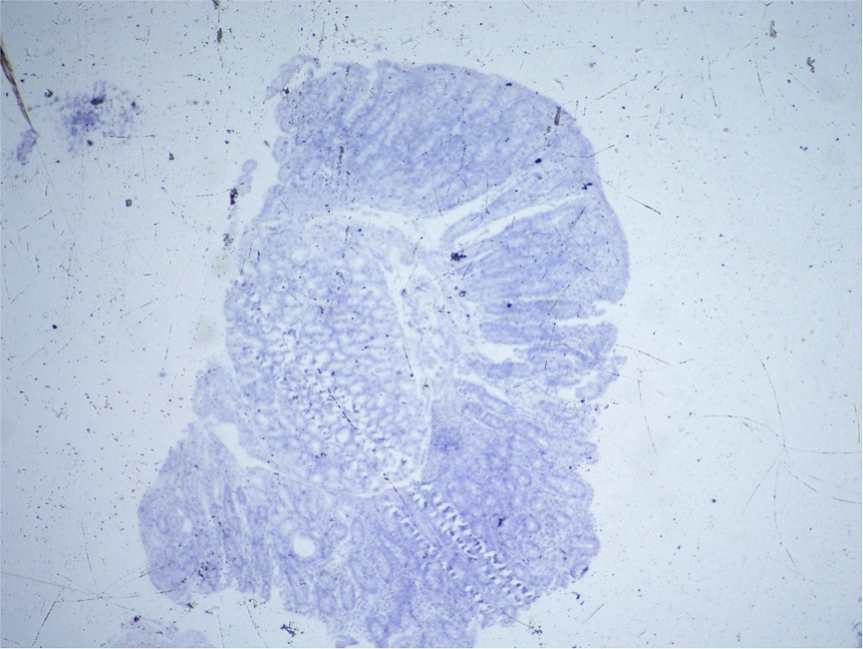
**The histopathology report of the small intestine showed severe villous atrophy grade IV with crypt hyperplasia consistent with celiac disease.** Old Marsh-Oberhuber classification: Type 3c: Total villous atrophy with completely flat mucosa and increased intraepithelial lymphocytes.

Initial laboratory investigations that were done are mentioned in Additional file 1: Table S1. X-rays of her upper and lower limbs showed diffuse osteopenia and bowing of both legs and forearms with blurring of the metaphyseal lines. It also showed dense transverse lines in tibia and ulna suggestive of looser’s zones indicative of severe rickets (Figures 2 and 3).Anti- endomysial antibodies titer was 80 (normal is negative), anti-tissue transglutaminase IgA was positive 75 U/ml (normal <2.5 U/ml) and anti-tissue transglutaminase IgG was negative. Upper endoscopy was done with small intestinal biopsy. The duodenum showed scalloping and fissuring of the small bowel suggestive of celiac disease. The histopathology report of the small intestine showed severe villous atrophy grade IV with crypt hyperplasia consistent with celiac disease. Old Marsh-Oberhuber classification: Type 3c: Total villous atrophy with completely flat mucosa and increased intraepithelial lymphocytes (Figure 4).

The patient was started on a gluten free diet. She also was started on Vitamin D supplements, calcium and iron replacement. The patient returned for follow-up 5 months later, she was feeling better, her weight had increased to 14 kg and her height had increased to 97 cm. She had taken iron and calcium supplements for a very short period but she did continue on a gluten free diet. The family was very poor and on further questioning on their dietary habit, it lacked many of the main constituents and was very low in vitamin D for most of her life but she did live in a very sunny area and before becoming crippled she had adequate sun exposure. Her diet before diagnosis was found to consist mainly of grains and breads with little protein and after being diagnosed and receiving instructions on a gluten free diet it was changed to rice and potatoes. Laboratory investigations showed some improvement from 5 months earlier these are shown in Additional file 1: Table S1. The patient was seen by an orthopedic surgeon who wanted the general condition of the patient to improve before considering any surgery.

The patient and her family were further instructed again on a gluten free diet emphasizing the available options. She was given further iron, calcium and Vitamin D replacement, the patient was not able to come back for follow up but 4 months later the family phoned and said she had markedly improved and had started walking.

## Discussion

There still remains a high rate of undiagnosed CD [1]. It affects 0.6 to 1% of the population worldwide. Measurement of IgA anti-tissue transglutaminase antibodies is the preferred initial screening test for CD because of its high sensitivity and specificity [1]. Screening should be done in patients with typical gastrointestinal symptoms suggestive of malabsorption, such as chronic diarrhea with weight loss, steatorrhea, postprandial abdominal pain and bloating [9] but should also be considered in patients with failure to thrive and those with metabolic bone disease according to NICE guidelines 2009 [10]. Several studies in Arab children have shown rickets to be quite common in children with CD [6–8].

A study in Saudi Arabia done on children with rickets showed that 38.4% of the children in the study were tested positive for celiac disease [6]. Another study In Jordan showed that 26% of the children with CD had rickets [7]. Another study in Kuwait showed that rickets occurred in about 25% of the children with CD [8]. There are no studies on vitamin D deficiency in Yemen but studies in neighbouring countries as Saudi Arabia, Lebanon and the United Arab Emirates reported that 30-65% of children and adults had 25-hydroxyvitamin D levels under 20 ng/dl [11–13]. Our patient showed other manifestations of a systemic disease as failure to thrive and anemia in addition to the severe rickets this should have triggered physicians to do further work up for a systemic disease causing malabsorption.

## Conclusion

Celiac disease can present with extraintestinal manifestations and it should be kept in mind in patients who present with failure to thrive and metabolic bone disease.

Rickets can be the first presentation of celiac disease and work up for celiac disease should be done in patients not responding to usual forms of treatment, or when associated with other manifestations suggestive of malabsorption. Our patient presented very late with severe deformities due to misdiagnosis and also poverty which caused a delay in seeking proper medical treatment. She was started on a gluten free diet which helped with the growth failure and anemia but she will need reconstructive surgery for her bone disease. Early diagnosis could have prevented these complications.

## Consent

Written informed consent was obtained from the patient’s legal guardian for publication of this case report and any accompanying images. A copy of the written consent is available for review by the Editor-in-Chief of this journal.

## Electronic supplementary material

Additional file 1: Table S1: Laboratory investigations done for the patient on her first and 2^nd^ visit to the hospital. (DOC 32 KB)

## Abbreviations

CD: Celiac disease.
