# Supplementary material for: Severe rickets in a young girl caused by celiac disease: the tragedy of delayed diagnosis: a case report
Source: BMC Res Notes. 2014 Oct 8;7:701. doi: 10.1186/1756-0500-7-701 (PMC4198728; doi:10.1186/1756-0500-7-701)
Supplement: Supplementary file 1 — Additional file 1: Table S1: Laboratory investigations done for the patient on her first and 2nd visit to the hospital. (DOC 32 KB) [file 13104_2014_3231_MOESM1_ESM.doc]

**Table 1** Laboratory investigations done for the patient on her first and 2^nd^ visit to the hospital

| Investigations | First visit | 2^nd^ visit (5 months later) | Normal range |
| --- | --- | --- | --- |
| Hemoglobin | 7.3 gm/dl | 9.8 gm/dl | 11.5-16.5gm/dl |
| MCV | 54.5 fl | 55.1 fl | 77-93 fl |
| calcium | 7.4 mg/dl | 7.6 mg/dl | 8.8-10.5 mg/dl |
| Creatinine | 0.27 mg/dl | Not available | <1.1 mg/dl |
| albumin | 3.3 gm/dl | 3.8 gm/dl | 3.4-4.8gm/dl |
| PTH | 98.2 pg/ml | 113 pg/ml | 7-65pg/ml |
| phosphorous | 1.47 mg/dl | 2.5 mg/dl | 2.5-5 mg/dl |
| Vitamin D | 14.4 ng/ml | 48.8 ng/ml | 30-40 ng/ml |
| Alkaline phosphatase | 458 u/L | 261 u/L | <240 u/L |
| SGPT | Not available | 125 U/L | Up to 55 U/L |
| Ferritin | Not available | 6.08 ng/ml | 7-142 ng/ml |
